# High-Temperature Structural and Electrical Properties of Ba*Ln*Co_2_O_6_ Positrodes

**DOI:** 10.3390/ma13184044

**Published:** 2020-09-11

**Authors:** Iga Szpunar, Ragnar Strandbakke, Magnus Helgerud Sørby, Sebastian Lech Wachowski, Maria Balaguer, Mateusz Tarach, José M. Serra, Agnieszka Witkowska, Ewa Dzik, Truls Norby, Maria Gazda, Aleksandra Mielewczyk-Gryń

**Affiliations:** 1 Nanotechnology Centre A, Faculty of Applied Physics and Mathematics and Advanced Materials Centre, Gdańsk University of Technology, ul. Narutowicza 11/12, 80-233 Gdańsk, Poland; sebastian.wachowski@pg.edu.pl (S.L.W.); agnieszka.witkowska@pg.edu.pl (A.W.); ewa.dzik@pg.edu.pl (E.D.); maria.gazda@pg.edu.pl (M.G.); 2 Department of Chemistry, Centre for Materials Science and Nanotechnology, University of Oslo, FERMiO, Gaustadalléen 21, NO-0349 Oslo, Norway; truls.norby@kjemi.uio.no; 3 Department for Neutron Materials Characterization, Institute for Energy Technology, Instituttveien 18, 2007 Kjeller, Norway; magnus.sorby@ife.no; 4 Instituto de Tecnología Química, Universitat Politècnica de València, Consejo Superior de Investigaciones Científicas, Av. Naranjos s/n, E-46022 Valencia, Spain; mabara@upvnet.upv.es (M.B.); mata8@itq.upv.es (M.T.); jmserra@itq.upv.es (J.M.S.)

**Keywords:** positrode, cobaltites, synchrotron powder diffraction, X-ray absorption spectroscopy, ceramics, thermal expansion, chemical expansion

## Abstract

The application of double perovskite cobaltites Ba*Ln*Co_2_O_6−δ_ (*Ln* = lanthanide element) in electrochemical devices for energy conversion requires control of their properties at operating conditions. This work presents a study of a series of Ba*Ln*Co_2_O_6−δ_ (*Ln* = La, Pr, Nd) with a focus on the evolution of structural and electrical properties with temperature. Symmetry, oxygen non-stoichiometry, and cobalt valence state have been examined by means of Synchrotron Radiation Powder X-ray Diffraction (SR-PXD), thermogravimetry (TG), and X-ray Absorption Spectroscopy (XAS). The results indicate that all three compositions maintain mainly orthorhombic structure from RT to 1000 °C. Chemical expansion from Co reduction and formation of oxygen vacancies is observed and characterized above 350 °C. Following XAS experiments, the high spin of Co was ascertained in the whole range of temperatures for BLC, BPC, and BNC.

## 1. Introduction

The double perovskite cobaltites Ba*Ln*Co_2_O_6-δ_ (where *Ln* is a lanthanide) have been a subject of research attention in recent years due to their transport properties and possible application as electrodes in electrochemical devices for energy conversion. The substantial number of oxygen vacancies in oxygen-deficient layers of *Ln*O_δ_ is believed to be beneficial for fast oxygen ion diffusion in these materials, while an overlap of Co3d and O2p orbitals in the Co-O slabs is favorable for enhanced electronic transport [1,2]. Furthermore, the flexibility of the double perovskite structure allows for substantial variations in oxygen non-stoichiometry and effective cobalt valence state, giving rise to high mixed ionic and electronic conductivity and fast surface kinetics [3].

In this study, we report the influence of temperature on structure and oxygen non-stoichiometry for a series of Ba*Ln*Co_2_O_6−δ_ compositions. High-temperature structural Synchrotron Radiation Powder X-ray Diffraction (SR-PXD) has been used to determine the temperature-dependent evolution of the materials’ structure accompanied by the oxygen loss due to reduction (). In combination with thermogravimetry (TG), thermal and chemical unit cell expansion has been established for BaLaCo_2_O_6−δ_ (BLC), BaPrCo_2_O_6−δ_ (BPC), and BaNdCo_2_O_6−δ_ (BNC). X-ray Absorption Spectroscopy (XAS) (room temperature) was undertaken for analysis of electronic states, with particular emphasis put on cobalt spin states analysis for BNC, BPC, and BLC.

## 2. Materials and Methods

The polycrystalline Ba*Ln*Co_2_O_6-δ_ samples used in this study were prepared through a conventional solid-state reaction. Powders of La_2_O_3_ (99.99% Alfa Aesar, Ward Hill, MA, USA, preheated at 900 °C for 5 h), Pr_6_O_11_ (99.99% Sigma-Aldrich, St. Louis, MO, USA), or Nd_2_O_3_ (99.9%, Chempur, preheated at 900 °C for 5 h) were used as lanthanide sources. Stoichiometric quantities of BaCO_3_ (99.9% Sigma-Aldrich), Co_2_O_3_ (99.8% Alfa Aesar), and the respective lanthanide oxide were ground in an agate mortar and pressed into pellets at 1.5 MPa. Pellets were then annealed at 1100 °C in static air for 48 h in a tube furnace at a heating/cooling rate of 2 °C/min. BaLaCo_2_O_6−δ_ was further annealed in Ar flow at 1050 °C for 24 h and in the airflow at 350 °C for 3 h to adopt a layered double perovskite structure.

Oxygen stoichiometry and oxidation state of cobalt were determined using iodometric titration at room temperature. Experimental procedure and details can be found elsewhere [4]. Approximately 15–20 mg of sample was used in this procedure.

Powder X-ray diffraction was used for the determination of sample purity, quality, and composition. X-ray diffractograms were in the 2θ angle range of 20 to 90° and were acquired using Philips X’Pert Pro diffractometer (Almelo, The Netherlands) with Cu Kα radiation. Samples were ground into powders and placed on the zero background slides for analysis.

The thermogravimetric oxidation studies were performed in synthetic air as purge gas (40 mL/min air purge gas, 20 mL/min protective N_2_) atmosphere using a Netzsch Tarsus 401 thermal analyser (Selb, Germany) in the temperature range RT-900 °C, with a temperature step of 2 °C/min on both cooling and heating.

High-temperature synchrotron powder X-ray diffraction (SR-PXRD) patterns were collected at the Elettra-Synchrotron Trieste, Trieste, Italy at the Materials Characterization by X-ray Diffraction (MCX) beamline. Diffraction patterns were collected at 20 KeV energy and 2θ angle range of 1 to 35° at from RT to 1000 °C in air, the heating rate between each temperature step 3 °C/min. Experimental data were analyzed by the Rietveld method using GSAS-II software (Argonne National Laboratory, Argonne, IL, USA) [5,6].

The combination of thermogravimetric oxidation studies and high-temperature synchrotron powder X-ray diffraction was used to determine the volumetric chemical expansion coefficient. Continuous equilibrium during heating and cooling was ensured by additional thermo-gravimetric temperature ramps, where heating and cooling rates of 2°/min were compared to 3°/min, thus eliminating any uncertainties due to different heating rates applied in the two techniques (Figure S1).

UV–Vis spectra of the powders were recorded on a Varian 5000 UV–Vis–NIR spectrophotometer (Varian, Inc., Palo Alto, CA, USA) in the range of 200 to 800 nm using BaSO_4_ as reference material and with a lamp change at 350 nm. The optical gap was then determined using the Kubelka–Munk theory.

X-ray Absorption Spectroscopy measurements were performed at the Solaris National Synchrotron Radiation Centre in Kraków, Poland. A dedicated PEEM/XAS bending magnet beamline was utilized to measure Re-M_4,5_, Co-L_2,3_, and O-K edges. Powders of samples were mounted on the carbon tape and placed on the Omicron plates for measurements.

## 3. Results and Discussion

**Figure 1:**
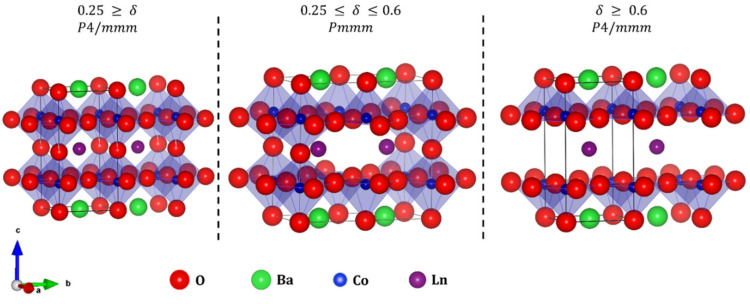
Unit cell of tetragonal (*P*4/*mmm*) and orthorhombic (*Pmmm*) BaLnCo_2_O_6-δ_ with the different oxygen content.

The structure of double perovskite cobaltites has been the subject of numerous studies [7,8,9,10,11,12]. Those materials are reported to adopt both tetragonal (*P*4/*mmm*) [9,10,13] and orthorhombic (*Pmmm*) [9,12] structures. The difference in tetragonal and orthorhombic unit cells results from oxygen vacancies ordering along the b-axis. Figure 1 presents the differences between tetragonal and orthorhombic structures with different oxygen content. The obtained crystal structure of Ba*Ln*Co_2_O_6−δ_ strongly depends on oxygen stoichiometry [4,7,8], as well as synthesis procedure [9]. As the difference between the two polymorphs, resulting from the oxygen vacancy ordering, is very subtle, the structural studies require very high-quality data such as synchrotron radiation X-ray powder diffraction collected with 2D detectors. Neutron diffraction, which is much more sensitive to oxygen, is a powerful complementary technique for determination of the oxygen vacancy concentrations at different sites [11,12,14,15,16,17,18,19].

**Figure 2:**
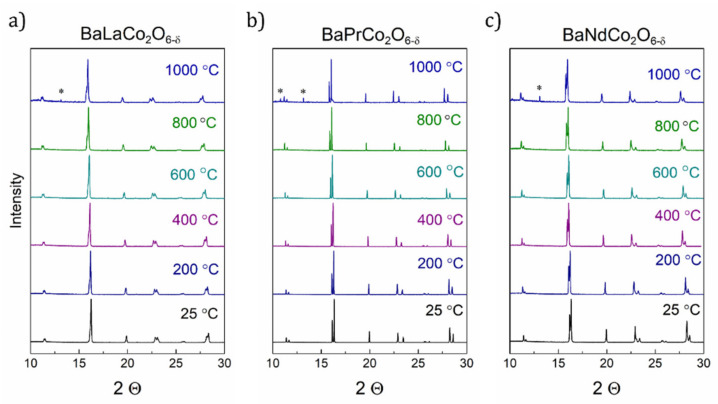
High-temperature Synchrotron Radiation Powder X-ray Diffraction (SR-PXD) diffractograms of BLC (**a**), BPC (**b**), and BNC (**c**). * denotes reflections from a reaction product between the sample and the silica capillary.

Figure 2 depicts the SR-PXD patterns of BLC, BPC, and BNC as a function of temperature. No phase transitions are observed in the interval RT-1000 °C. The shift of peak positions towards lower angles is due to the chemical and thermal expansion. Rietveld refinements were performed with orthorhombic *Pmmm* structures (a_p_ × 2a_p_ × 2a_p_) for all three compositions. The orthorhombic reflections are very subtle and hard to detect using methods basing on X-ray radiation. However, in our previous study [12], we showed by the combined use of SR-XRD and neutron diffraction that BLC, BPC, and BNC adopt orthorhombic symmetry at room temperature. Therefore, even though higher symmetry refinement (*P*4/*mmm*) is possible for BNC in this study, we follow the previous refinements of higher quality data and ascribe orthorhombic structure also to BNC. BLC and BNC showed additional minority phases of, respectively, tetragonal (*P*4/*mmm*) and cubic ($Pm\bar{3}m)$ symmetry.

**Figure 3:**
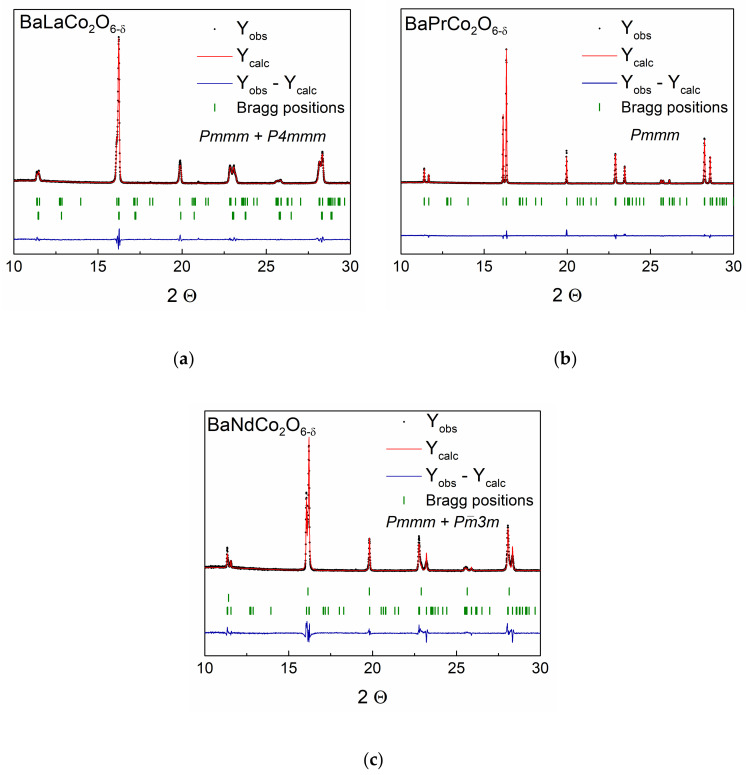
Observed (dotted lines), calculated (solid lines), and difference (bottom) SR-PXD for BLC (**a**), BPC (**b**), and BNC (**c**) at RT.

Figure 3 presents the room temperature SR-PXD diffraction patterns with Rietveld refinement profiles for the three compositions. The detailed results of Rietveld refinement are collected in Supplementary Information (Tables S1–S27). The lattice parameter doubling along the *c*-axis confirms *A*-site cation ordering, while the double *b*-parameter results from oxygen vacancy ordering [12]. BPC has been reported to form both tetragonal and orthorhombic structure, strongly depending on oxygen stoichiometry [4,6,7,8,20], as well as synthesis procedure [21], but is reported to be stabilized in orthorhombic structure when oxygen non-stoichiometry (δ) is between 0.25 and 0.6 [14].

In this study, δ for the orthorhombic BPC structure was 0.36, according to iodometric titration results at room temperature, which is in agreement with the previous reports [20,22,23].

**Figure 4:**
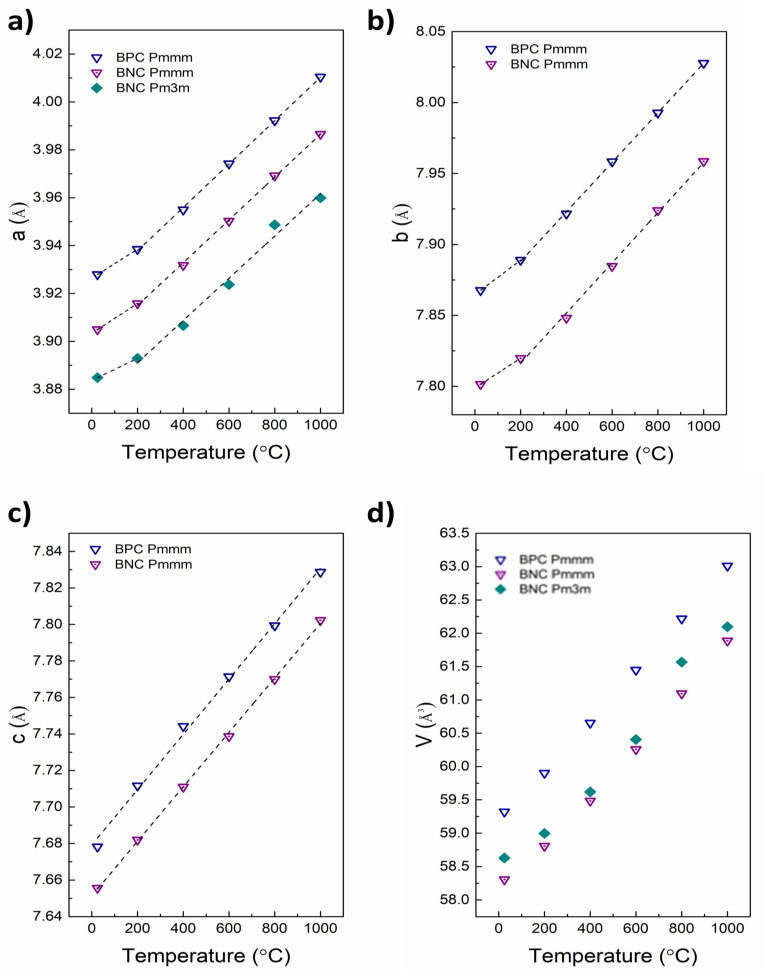
Temperature dependence of lattice parameters and unit cell volume of BPC and BNC. *a*-parameter (**a**) *b*-parameter (**b**) *c*-axis (**c**) unit cell volume (**d**).

The temperature evolution of the lattice parameters for BNC and BPC is presented in Figure 4. We have kept the orthorhombic structure as refinement basis over the whole temperature range for all three compositions given our background data [12], and the fact that the orthorhombic phase for BNC and BPC is generally reported to be stabilized in 0.25 ≤ δ ≤ 0.6, while the tetragonal structure is adopted if the oxygen content is lower than 5.4, or higher than 5.75 [13,14,16,23]. Following this, and given that a ≠ b/2 at all temperatures (Figure S2), our data do not support any phase transition between RT and 1000 °C for any of the compositions.

The lattice parameters and the cell volumes increase linearly, but with different slopes below and above approximately 350 °C for the *a*- and *b*-parameter, where a combination of thermal and chemical expansion can be seen, as expected for reducible metal-based mixed conducting oxides [24]. The thermal expansion is related only to the inherent vibrational properties of the crystal lattice, while the chemical expansion of the *ab*-plane results from the increasing concentration of oxygen vacancies [24]. Oxidation studies of all three compositions show that δ changes very little between RT and approximately 300 °C, following the literature [16]. Table 1 reports the values of the linear expansion coefficient in two separate temperature ranges calculated as a slope of relation presented in Figure 4 for particular unit cell parameters. The lower temperature value (up to 200 °C) is a product of thermal expansion, while for higher temperature (above 200 °C) it is a combination of thermal and chemical expansion.

**Figure 5:**
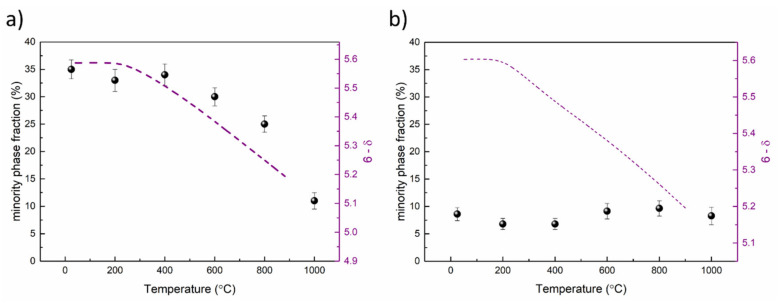
The fraction of the minority phases (dot) P4/mmm for BLC (**a**) and Pm3 m for BNC (**b**), and oxygen stoichiometry (dashed line) as a function of temperature. The oxygen stoichiometry changes were calculated based on thermogravimetry using titration results as a starting point.

**Figure 6:**
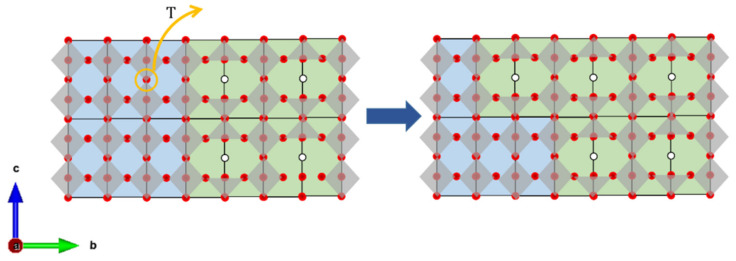
The scheme of the phase transition from tetragonal (blue unit cells) to orthorhombic (green unit cells) phase.

The fractions of majority *Pmmm* and minority *P*4/*mmm* phases in BLC changes with temperature (Figure 5a). As the temperature increases, a reduction in the tetragonal phase fraction is observed, which can be correlated with oxygen loss. Oxygen vacancy ordering increases with increasing oxygen non-stoichiometry [16], gradually turning *P*4*/mmm* into *Pmmm* by ordering oxygen vacancies along the *b-*axis. As the orthorhombic phase for BNC and BPC is generally stabilized in 0.25 ≤ δ ≤ 0.6, the tetragonal structure is adapted if the oxygen content is lower than 5.4, or higher than 5.75 [13,14,16]. The scheme of the phase transition from tetragonal to the orthorhombic structure is presented in Figure 6. Oxygen vacancy formation in the tetragonal phase leads to the transformation of a particular unit cell to the orthorhombic structure, increasing the majority phase content. The oxygen content in the remaining tetragonal phase is thus constant, although oxygen is released simultaneously from both phases.

Such a relation between oxygen stoichiometry and phase composition was not observed for BNC, where a minority cubic phase fraction of ~7% was constant in the measured T-range (Figure 5b).

**Figure 7:**
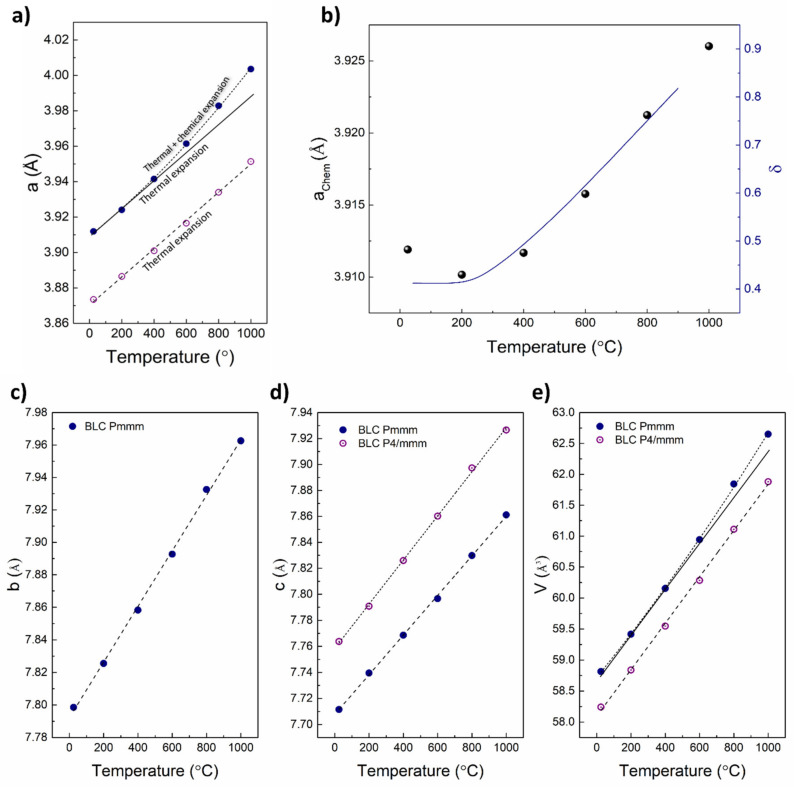
Temperature evolution of lattice parameters of orthorhombic and tetragonal BLC (**a**,**c**–**e**). Correlation between change in a parameter, resulting from chemical expansion, and the concentration of oxygen vacancies (**b**).

Oxygen loss in tetragonal BLC leads to a phase transition to the orthorhombic structure. The temperature evolution of lattice parameters (Figure 7) suggests that oxygen stoichiometry in the remaining tetragonal phase is constant in the whole temperature range. The *a*-parameter of tetragonal BLC increases linearly, while in the orthorhombic phase deviates upwards from linear relation, which is typical of chemical expansion upon reduction and confirmed by the dependence on the increasing concentration of oxygen vacancies (Figure 7b) [24]. The temperature dependence of the two-unit cell volumes shows a similar behavior as the *a*-parameters, being linear for the tetragonal phase and nonlinear for the orthorhombic one. Interestingly, both the *c-* and *b*-parameters of orthorhombic BLC change linearly with temperature, indicating anisotropic chemical expansion.

**Figure 8:**
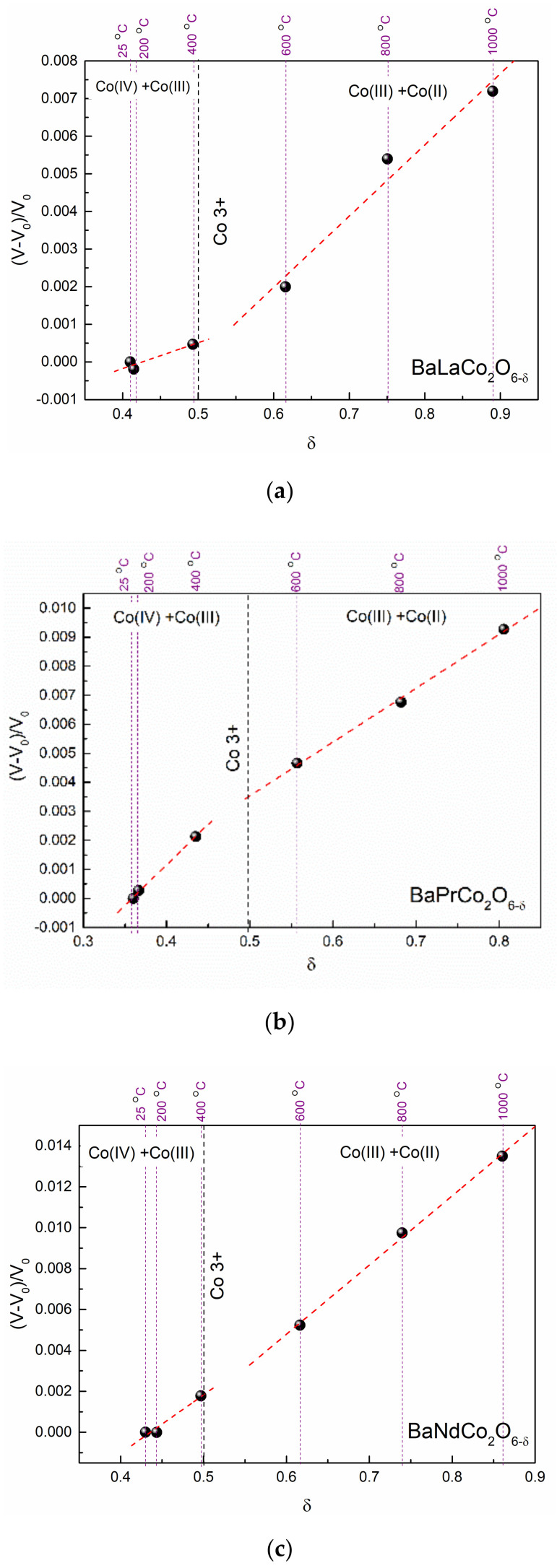
Relative change of unit cell volume from the chemical expansion of the orthorhombic phase as a function of oxygen non-stoichiometry for BLC (**a**), BPC (**b**), and BNC (**c**). Thermal expansion is subtracted.

Figure 8 depicts the chemical expansion of BLC, BNC, and BPC as a relative change in orthorhombic unit cell volume vs. oxygen non-stoichiometry. The change due to the thermal expansion was subtracted from the total volume, thus the presented changes result only from the chemical expansion of the orthorhombic phase.

As can be seen from Figure 4 and Figure 7, the chemical expansion is anisotropic. The slope change upon oxygen loss is observed only along the *a*- and *b-*axes for BPC and BNC, and along the *a*-axis alone for BLC. The analysis of directional chemical expansion requires information of anisotropic shape and size of the cobalt ions, and localization of oxygen vacancies. In this study, the chemical expansion is analyzed non-directionally and given as total volumetric chemical expansion. The data was divided into two regions, where different chemical expansion models were applied. The chemical expansion results from both formation of oxygen vacancies and reduction of cobalt ions. Due to the different ionic sizes of Co^2+^, Co^3+^, and Co^4+^, the chemical expansion in the two regimes should be described separately. However, in both cases, the chemical reaction driving the chemical expansion is oxygen exchange. Therefore, the chemical expansion coefficient (*β*) always consists of two parts: one originating from the formation of oxygen vacancies ($\beta_{V_{O}^{••}})\;$ and the second from cobalt reduction ($\beta_{Co}$) (Equation (1)). The *β_Co_* is doubled because there are two reduced cobalt ions per one oxygen vacancy. The total chemical expansion coefficient upon reduction can be determined experimentally, analyzing the temperature evolution of unit cell volume minus the effect of thermal expansion. Then, two components of chemical expansion can be considered separately.

(1) $$\beta =\;\beta_{V_{O}^{••}}+2\;\beta_{Co}$$

The obtained values of the chemical expansion coefficients are given in Table 2. The derivations are given in the Appendix A.

As shown, Co^3+^ exhibits a high spin (HS) state in BLC. This is in line with previous studies [23,25] stating that the HS of Co^3+^ and Co^2+^ is energetically more favorable. Thus, the ionic radii for cobalt at HS were used for calculations, with values of 0.53 Å, 0.61 Å, and 0.745 Å for Co^4+^, Co^3+^, and Co^2+^, respectively [26,27]. In both regimes, the chemical expansion coefficient *β_red_* is positive, meaning that the reduced oxygen content leads to unit cell volume increase. However, the total chemical expansion is a result of two separate effects. The reduction of cobalt ions gives a positive contribution to the chemical expansion. The negative value of the chemical expansion coefficient related to oxygen vacancy formation gives the information that the oxygen vacancy formation itself leads to the unit cell contraction.

Comparable values of the chemical expansion coefficient were reported in previous studies on perovskite oxides [19,28,29,30]. The most studied system is La_1−x_Sr_x_Co_y_Fe_1−y_O_3−δ_ (LSCF) [24], where the total chemical expansion coefficient ranges from 0.022 for x = 0.4 and y = 0.8 to 0.059 for x = 0.5 and y = 0. The reported values refer to high temperatures, thus it should be compared to the regime δ > 0.5.

The approach of separating the effect of cation reduction and oxygen vacancy formation on total expansion is still uncommon and based mostly on DFT studies, but the available studies confirm that the oxygen vacancies cause unit cell contraction [24]. The $\beta_{v_{O}^{••}}$ significantly differs in the two investigated regimes. The determined values of $\beta_{v_{O}^{••}}$ were used to calculate the volume of oxygen vacancy, according to Equation (A27) in the Appendix A. In this case, the oxygen vacancy size is a measure of lattice deformation.

**Figure 9:**
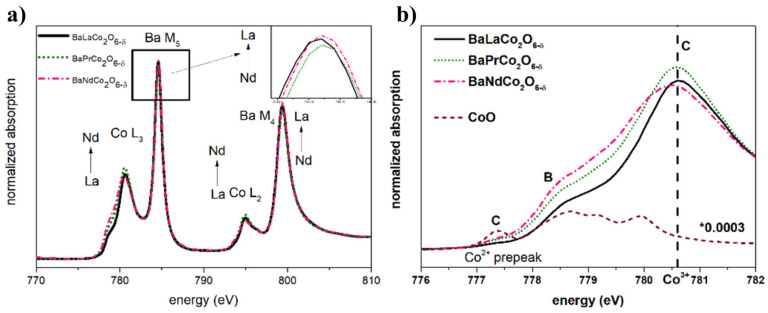
(**a**) X-ray Absorption Spectroscopy (XAS) spectrum of Co L_2,3_ and Ba M_4,5_ edges. (**b**) XANES (X-ray Absorption Near Edge Structure) spectra of Co L_3_-edge for studied materials and reference sample (CoO).

**Figure 10:**
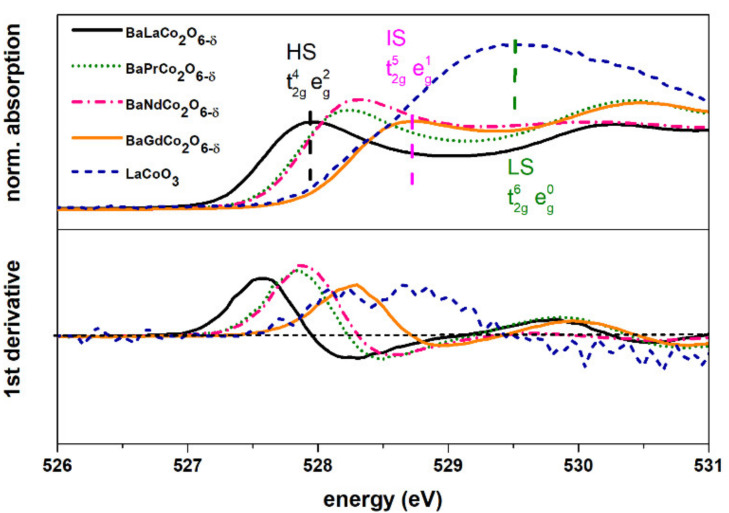
X-ray absorption spectra of oxygen K-edge in the Co 3d bands region. For comparison, intermediate spin (BaGdCo_2_O_6_) and low spin (LaCoO_3_) reference samples are included.

**Figure 11:**
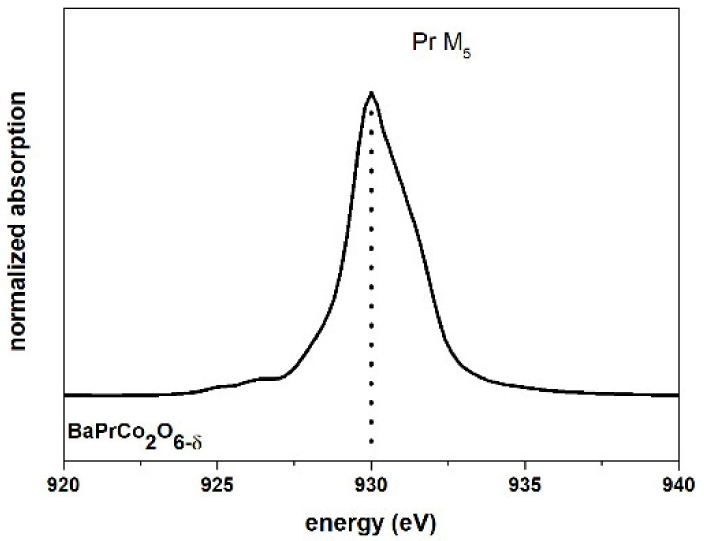
XANES praseodymium M_5_ edge for BaPrCo_2_O_6_.

Figure 9, Figure 10 and Figure 11 present the results of the X-ray absorption studies (XAS) of the as-prepared samples at room temperature for the Co L_2,3_-edges, Ba M4,5-edges, O K-edge, and Pr M_4,5_-edges spectra, respectively. Figure 7a shows the XAS data for Co L_2,3_ and Ba M_4,5_ edges collected for all studied compounds and reference samples. The intensity of the white lines (WL) attributed to both cobalt and barium orbitals varies between compositions. With the decrease in ionic radius from lanthanum (1.172 Å) to gadolinium (1.078 Å) (for six-fold coordination [25,26]), the WL of cobalt increases while barium WL intensity decreases, indicating that the decrease of the ionic radius is causing an increase in the density of the unoccupied electron 3d Co states and a decrease in the density of unoccupied electronic 4f Ba states. This relation is accompanied by the differences observed on the lower energy slope of Co L-edges (especially of L_3_-edge).

Figure 9 presents the Co L_3_-edge (normalized XANES) for all investigated compositions along with the reference (CoO). The reference CoO spectrum has been scaled down to highlight the correlation between the pre-edge structure feature (A and B) and edge intensity (C). An almost undetectable pre-peak (A) in the spectra of the investigated samples suggests a negligible concentration of Co^2+^ species. The main line attributed to Co^3+^ (C) is present at the same energy for BLC, BPC, and BNC (780.6 eV). Note that there are no significant differences in the oxygen non-stoichiometry of the measured samples (Table 3).

The shoulder on B—reflecting Co^2+^—is visible for all samples; however, it is more extensive for BPC and BNC than for BLC. This may suggest that the more oxidized BLC—with more octahedrally coordinated Co—exhibits a more HS character. Such a relation has been previously reported for samples with fixed δ = 0.5 [31]. The complementary to XAS cobalt L-edges data is oxygen K-edge analysis (Figure 10).

Oxygen K-edge is sensitive to the density of empty cobalt 3d t_2g_ and e_g_ states through hybridization with oxygen 2p orbitals. LaCoO_3_ has been chosen as a reference for the analysis of O K-edge because it contains solely Co^3+^ in a low spin (LS) state (t_2g_^6^ e_g_^0^, S = 0) [32] along with BaGdCo_2_O_6_ which was previously reported to exhibit IS [31]. The comparison of recorded spectra supports the analysis of cobalt L-edges. Evaluation of the pre-peak position (between 526 and 531 eV) and comparison with the references reveals the highest density of 3d t_2g_ empty states (in the octahedral environment) for the BLC sample. On the other hand, the BGC spectra indicate a lower density of unoccupied t_2g_ state in comparison to the samples with larger lanthanides, which is partially caused by higher oxygen non-stoichiometry and the lower ionic radius of Gd with respect to La, Pr, and Nd. These results suggest that in the cobaltites with Pr and Nd, mixed HS/IS state of cobalt has been detected. This means that in the case of BLC exhibiting HS, the crystal field splitting is lower than in the case of BPC and BNC.

Figure 11 presents the Pr M_5_ edge recorded for the BPC sample. The WL position for this spectrum (930 eV) suggests the dominance of Pr^3+^. Herrero-Martin et al. reported the WL shift towards lower energies for higher Pr^3+^ content relative to Pr^4+^. In their simulation, the WL peak for 85:15 Pr^3+^ to Pr^4+^ content in Pr_0.5_Ca_0.5_CoO_3_ should be observed for ~934 eV, while the experimental data for Pr_2_O_3_ shows the WL at 931 eV [33].

**Figure 12:**
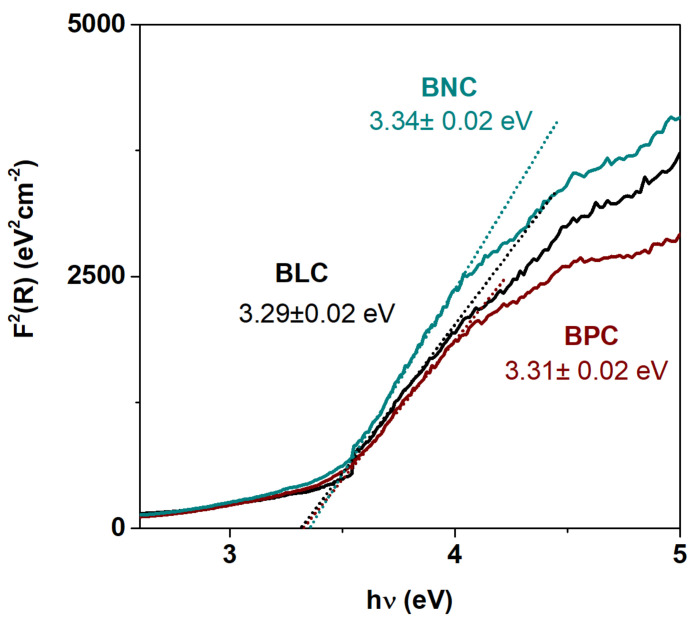
Plot of Kubelka–Munk function vs. energy for all measured compositions as well as values of energy gap at RT for BLC, BPC, and BNC.

UV–Vis (Figure 12) absorption experiments were performed to study the influence of the lanthanide dopant on the bandgap. The bandgap normally refers to the energy difference between valence and conduction bands. The optical band-gap energy is thus normally comparable to the thermal bandgap related to the formation of electron–hole pairs. Such an intrinsic formation of electrons and holes is the Co charge disproportionation reaction:

(2) $$2Co^{3+}\to Co^{2+}+Co^{4+}$$

The Kubelka–Munk function as given below was used to determine the optical band gap of investigated materials.

(3) $$F\left(R\right)=\frac{{\left(1-R\right)}^{2}}{2R}$$

where *R* is diffuse reflectance emanating from an infinitely thick sample [34].

This evaluation involves the plotting of the obtained (hνF(*R*N)^2^) as a function of hν. The bandgap E_g_ can be obtained by extrapolating a tangent line drawn in the point of inflexion of the curve to zero, i.e., the point of intersection with the hν horizontal axis. Figure 8 shows the room temperature UV–Vis absorption spectra of the BLC, BNC, and BPC.

As observed, the lanthanide is not causing any shift of the absorption edge. The optical bandgap values for all investigated samples are similar and around 3.3 eV within the error range. The obtained values for the optical band gap are surprisingly high for the black powders. However, as the investigated materials may be considered as degenerate semiconductors, their color can be a result of either intra-band electronic transition or the transitions related to the in-gap states presented. It is reported that electrons and holes are transferred in a partially filled, degenerate O 2p Co 3d band, and that it is thus located at the top of the valence band. The measured optical bandgap may therefore not represent the electrical band-gap. The presence of band states within the bandgap of partially filled anti-bonding σ *-bands, wherein electronic conduction can occur, is previously reported for double perovskite cobaltites [35,36,37].

## 4. Conclusions

We have investigated the electrical and structural properties of chosen Ba*Ln*Co_2_O_6−δ_ (*Ln* = La, Pr, and Nd) double perovskites. All measured compositions (BLC, BNC, and BPC) were refined to orthorhombic (*Pmmm*) structure up to 1000 °C. Moreover, BLC and BNC showed additional tetragonal (*P*4/*mmm*) and cubic ($Pm\bar{3}m)$ minority phases, respectively. The thermal evolution of the unit cells shows that after subtracting expansion from thermal lattice vibrations, partial reduction of cobalt and formation of oxygen vacancies gives positive and negative contributions, respectively, to the chemical expansion. The spectroscopic studies show that cobalt is present only in the intermediate or high-spin state for all compositions at room temperature. The optical bandgap is characterized, showing values of ~3.3 eV, which is not consistent with high electronic conductivity. We ascribe this to partially filled antibonding states at the valence band maximum with high mobility for electrons and electron holes [36,37].

## Figures and Tables

**Table 1:** Thermal expansion coefficients.

Material (Direction)	Expansion Coefficient (below 200 °C) (×10^−6^)	Expansion Coefficient (above 200 °C) (×10^−6^)
BPC (a)	15.3	23.0
BPC (b)	15.5	22.1
BPC (c)	19.7	
BNC *Pmmm* (a)	15.9	22.8
BNC *Pmmm* (b)	13.4	22.6
BNC *Pmmm* (c)	19.5	
BNC *Pm3m* (a)	11.9	22.6

**Table 2:** Chemical expansion coefficients for BLC, BPC, and BNC.

Material	Oxidation Range	$\beta_{red}$	$\beta_{Co}$	$\beta_{v_{O}^{••}}$
BLC	δ < 0.5	$\beta_{red}=0.007$	$\beta_{Co\;4\to 3}=0.355$	$\beta_{v_{O}^{••}}=-0.703$
	δ > 0.5	$\beta_{red}=0.019$	$\beta_{Co\;3\to 2}=0.822$	$\beta_{v_{O}^{••}}=-1.624$
BNC	δ < 0.5	$\beta_{red}=0.029$	$\beta_{Co\;4\to 3}=0.353$	$\beta_{v_{O}^{••}}=-0.677$
	δ > 0.5	$\beta_{red}=0.034$	$\beta_{Co\;3\to 2}=0.822$	$\beta_{v_{O}^{••}}=-1.610$
BPC	δ < 0.5	$\beta_{red}=0.028$	$\beta_{Co\;4\to 3}=0.361$	$\beta_{v_{O}^{••}}=-0.694$
	δ > 0.5	$\beta_{red}=0.019$	$\beta_{Co\;3\to 2}=0.822$	$\beta_{v_{O}^{••}}=-1.625$

**Table 3:** Co average oxidation state and oxygen non-stoichiometry obtained with iodometric titration BLC, BPC, and BNC for the samples before XAS studies.

Nominal Composition	Co Average Oxidation State	Oxygen Non-Stoichiometry
BaLaCo_2_O_6_	3.2880	0.2100
BaPrCo_2_O_6_	3.2540	0.2460
BaNdCo_2_O_6_	3.2332	0.2668

## Supplementary Materials

The following are available online at https://www.mdpi.com/1996-1944/13/18/4044/s1, Figure S1: The temperature evolution of oxygen stoichiometry in BaGdCo_2_O_6−δ_ with different heating rates, Figure S2: The temperature evolution of unit cell parameters a and b/2, Table S1: BaLaCo_2_O_6−δ_ goodness of fit, Table S2: Atomic coordinates and U_iso_ refined for orthorhombic phase of BaLaCo_2_O_6−δ_. At room temperature, Table S3: Atomic coordinates and U_iso_ refined for orthorhombic phase of BaLaCo_2_O_6−δ_. At 200 °C, Table S4: Atomic coordinates and U_iso_ refined for orthorhombic phase of BaLaCo_2_O_6−δ_. At 400°C, Table S5: Atomic coordinates and U_iso_ refined for orthorhombic phase of BaLaCo_2_O_6−δ_. At 600 °C, Table S6: Atomic coordinates and U_iso_ refined for orthorhombic phase of BaLaCo_2_O_6−δ_. At 800°C, Table S7: Atomic coordinates and U_iso_ refined for orthorhombic phase of BaLaCo_2_O_6−δ_. At 1000 °C, Table S8: Atomic coordinates and U_iso_ refined for tetragonal phase of BaLaCo_2_O_6−δ_ at room temperature, Table S9: Atomic coordinates and U_iso_ refined for tetragonal phase of BaLaCo_2_O_6−δ_ at 200 °C, Table S10: Atomic coordinates and U_iso_ refined for tetragonal phase of BaLaCo_2_O_6−δ_ at 400 °C, Table S11: Atomic coordinates and U_iso_ refined for tetragonal phase of BaLaCo_2_O_6−δ_ at 600 °C, Table S12: Atomic coordinates and U_iso_ refined for tetragonal phase of BaLaCo_2_O_6−δ_ at 800 °C, Table S13: Atomic coordinates and U_iso_ refined for tetragonal phase of BaLaCo_2_O_6−δ_ at 1000 °C, Table S14: BaPrCo_2_O_6−δ_ goodness of fit, Table S15: Atomic coordinates and U_iso_ refined for orthorhombic phase of BaPrCo_2_O_6−δ_ at room temperature, Table S16: Atomic coordinates and U_iso_ refined for orthorhombic phase of BaPrCo_2_O_6−δ_ at 200 °C, Table S17: Atomic coordinates and U_iso_ refined for orthorhombic phase of BaPrCo_2_O_6−δ_ at 400 °C, Table S18: Atomic coordinates and U_iso_ refined for orthorhombic phase of BaPrCo_2_O_6−δ_ at 600 °C, Table S19: Atomic coordinates and U_iso_ refined for orthorhombic phase of BaPrCo_2_O_6−δ_ at 800 °C, Table S20: Atomic coordinates and U_iso_ refined for orthorhombic phase of BaPrCo_2_O_6−δ_ at 1000 °C, Table S21: BaNdCo_2_O_6−δ_ goodness of fit, Table S22: Atomic coordinates and U_iso_ refined for orthorhombic phase of BaNdCo_2_O_6−δ_ at room temperature, Table S23: Atomic coordinates and U_iso_ refined for orthorhombic phase of BaNdCo_2_O_6−δ_ at 200 °C, Table S24: Atomic coordinates and U_iso_ refined for orthorhombic phase of BaNdCo_2_O_6−δ_ at 400 °C, Table S25: Atomic coordinates and U_iso_ refined for orthorhombic phase of BaNdCo_2_O_6−δ_ at 600 °C, Table S26: Atomic coordinates and U_iso_ refined for orthorhombic phase of BaNdCo_2_O_6−δ_ at 800 °C, Table S27: Atomic coordinates and U_iso_ refined for orthorhombic phase of BaNdCo_2_O_6−δ_ at 1000 °C.

## Appendix A

### Appendix A.1. Chemical Expansion of Cobalt Ions upon Reduction

Using a Kröger–Vink-compatible notation, the following description of Co ions can be introduced (Table A1) [35]:

**Table A1:** Cobalt ions notation.

Ion	Notation
$Co^{3.5+}$	$Co_{Co}^{X}$
$Co^{4+}$	$Co_{Co}^{\frac{1}{2}\;•}$
$Co^{3+}$	$Co_{Co}^{\frac{1}{2}\;/}$
$Co^{2+}$	$Co_{Co}^{\frac{3}{2}\;/}$

Two reaction equations linking the reduction of cobalt and the formation of oxygen vacancies can be written. One would be the reduction of *Co*^4+^ to *Co*^3+^ associated with vacancy formation (Equation (A1)), and the second can be formulated similarly for the reduction of *Co*^3+^ to *Co*^2+^ (Equation (A2)).

(A1) $$2\;Co_{Co}^{\frac{1}{2}\;•}+\;O_{O}^{X}\;\to \;2\;Co_{Co}^{\frac{1}{2}\;/}+\;v_{O}^{••}+\;\frac{1}{2}O_{2\;\left(g\right)}$$

(A2) $$2\;Co_{Co}^{\frac{1}{2}\;/}+\;O_{O}^{X}\;\to \;2\;Co_{Co}^{\frac{3}{2}\;/}+\;v_{O}^{••}+\;\frac{1}{2}O_{2\;\left(g\right)}$$

The regimes can be distinguished with regard to the presence of Co oxidation states in the material: oxidized state for *δ* < 0.5 and reduced state for *δ* > 0.5. In the former $\left[Co_{Co}^{\frac{1}{2}\;•}\right]+\;\left[Co_{Co}^{\frac{1}{2}\;/}\right]\gg \left[Co_{Co}^{\frac{3}{2}\;/}\right]$ and in the latter $\left[Co_{Co}^{\frac{1}{2}\;/}\right]+\;\left[Co_{Co}^{\frac{3}{2}\;/}\right]\gg \left[Co_{Co}^{\frac{1}{2}\;•}\right]$. The transition value of *δ* is 0.5, where the average cobalt oxidation state is 3.0.

The chemical expansion of cobalt results from the difference in size between cobalt oxidation states, and it is defined as

(A3) $$\beta_{Co}=\;\frac{1}{\Delta \delta }\;\cdot \;\frac{\Delta V_{Co}}{V_{Co_{0}}}$$

where Δ*δ* denotes the change of oxygen stoichiometry, which can be detailed as

(A4) $$\beta_{Co}=\;\frac{1}{\delta -\delta_{0}}\;\cdot \;\frac{V_{Co\left(\delta \right)}-V_{Co_{0}}}{V_{Co_{0}}}$$

Here, $V_{Co\left(\delta \right)}$ is the average volume occupied by Co at any given *δ* and $V_{Co_{0}}$ is the average volume occupied by Co at room temperature ($\delta_{0}$).

### Appendix A.2. Reduction from Co^4+^ to Co^3+^

The average cobalt oxidation state ($Co^{AVG}$) can be calculated from

(A5) $$2Co^{AVG}=2\left[Co_{Co}^{\frac{3}{2}\;/}\right]+3\left[Co_{Co}^{\frac{1}{2}\;/}\right]+4\left[Co_{Co}^{\frac{1}{2}\;•}\right]$$

In the oxidized regime, this gives

(A6) $$Co^{AVG}=\frac{3}{2}\left[Co_{Co}^{\frac{1}{2}\;/}\right]+2\left[Co_{Co}^{\frac{1}{2}\;•}\right]$$

$Co^{AVG}$ can be related to oxygen non-stoichiometry according to

(A7) $$Co^{AVG}=3.5-\;\delta$$

The concentration of cobalt ions in the oxidized regime is given by

(A8) $$\left[Co_{Co}^{\frac{1}{2}\;•}\right]+\left[Co_{Co}^{\frac{1}{2}\;/}\right]=2$$

The combination of Equations (A6)–(A8) gives the relations between the concentration of *Co*^3+^ and *Co*^4+^ with oxygen non-stoichiometry (Equation (A9)) in the oxidized regime.

(A9) $$\left[Co_{Co}^{\frac{1}{2}\;•}\right]=1-2\delta$$

(A10) $$\left[Co_{Co}^{\frac{1}{2}\;/}\right]=2\delta +1$$

The volume of cobalt can be described as the weighted arithmetic mean of *Co*^4+^ and *Co*^3+^ volume, where the concentration of each species is a weight (Equation (A11)).

(A11) $$V_{Co}=\;\frac{\left[Co_{Co}^{\frac{1}{2}\;•}\right]\cdot V_{Co^{4+}}+\left[Co_{Co}^{\frac{1}{2}\;/}\right]V_{Co^{3+}}}{2}$$

The average cobalt volume as a function of *δ* (Equation (A10)) can be obtained by including the Equations (A9) and (A10) to the Equation (A11).

(A12) $$V_{Co}\left(\delta \right)=\;\frac{\left(1-2\delta \right)\cdot V_{Co^{4+}}+\left(2\delta +1\right)\cdot V_{Co^{3+}}}{2}$$

Similarly, the average volume occupied by Co at room temperature relates to $\delta_{0}$:

(A13) $$V_{Co_{0}}=\;\frac{\left(1-2\delta_{0}\right)\cdot V_{Co^{4+}}+\left(2\delta_{0}+1\right)\cdot V_{Co^{3+}}}{2}$$

Inserting Equations (A12) and (A13) into Equation (A5) leads to the expression on chemical expansion coefficient of *Co*^4+^ to *Co*^3+^ reduction (Equations (A14) and (A15)),

(A14) $$\beta_{Co\;4\to 3}=\;\frac{1}{\Delta \delta }\cdot \frac{2\left(\left(\delta_{0}-\delta \right)\cdot V_{Co^{4+}}+\left(\delta -\delta_{0}\right)\cdot V_{Co^{3+}}\right)}{\left(1-\;2\delta_{0}\right)\cdot V_{Co^{4+}}+\left(2\delta +1\right)\cdot V_{Co^{3+}}}$$

(A15) $$\beta_{Co\;4\to 3}=\;\frac{2\cdot \left(V_{Co^{3+}}-V_{Co^{4+}}\right)}{\left(1-\;2\delta_{0}\right)\cdot V_{Co^{4+}}+\left(2\delta_{0}+1\right)\cdot V_{Co^{3+}}}$$

Cobalt volume can be calculated as sphere volume, leading to the Equation (A16).

(A16) $$\beta_{Co\;4\to 3}=\;\frac{2\cdot \left(r_{Co^{3+}}^{3}-r_{Co^{4+}}^{3}\right)}{\left(1-\;2\delta_{0}\right)\cdot r_{Co^{4+}}^{3}+\left(2\delta_{0}+1\right)\cdot r_{Co^{3+}}^{3}}$$

### Appendix A.3. Reduction of Co^3+^ to Co^2+^

The analogous consideration can be made in the reduced regime. The average cobalt oxidation state is given with Equation (A17) and cobalt ion concentrations in the reduced regime are given by Equation (A18).

(A17) $$Co^{AVG}=\frac{3}{2}\;\cdot \;\left[Co_{Co}^{\frac{1}{2}\;/}\right]+\;\left[Co_{Co}^{\frac{3}{2}\;/}\right]\;$$

(A18) $$\left[Co_{Co}^{\frac{1}{2}\;/}\right]+\left[Co_{Co}^{\frac{3}{2}\;/}\right]=2$$

The relation between the concentration of cobalt species with *δ* is given by Equations (A19) and (A20).

(A19) $$\left[Co_{Co}^{\frac{1}{2}\;/}\right]=3-2\delta$$

(A20) $$\left[Co_{Co}^{\frac{3}{2}\;/}\right]=2\delta -1$$

In the reduced state, the cobalt volume is also a weighted mean of *Co*^3+^ and *Co*^2+^ volume (Equation (A21)).

(A21) $$V_{Co}=\;\frac{\left[Co_{Co}^{\frac{3}{2}\;/}\right]\cdot V_{Co^{3+}}+\left[Co_{Co}^{\frac{3}{2}\;/}\right]V_{Co^{2+}}}{2}$$

Including Equations (A19) and (A20) to Equations (A21) and (A22) gives the relation between cobalt average volume and oxygen non-stoichiometry.

(A22) $$V_{Co}\left(\delta \right)=\;\frac{\left(3-2\delta \right)\cdot V_{Co^{3+}}+\left(2\delta -1\right)\cdot V_{Co^{2+}}}{2}$$

With the Equation (A21) the chemical expansion coefficient of *Co*^3+^ to *Co*^2+^ reduction can be calculated:

(A23) $$\beta_{Co\;3\to 2}=\;\frac{1}{\delta -\delta_{0}}\;\cdot \;\frac{\Delta V_{Co}}{V_{Co_{0}}}$$

In this regime, *δ*_0_ = 0.5 and $V_{Co_{0}}=\;V_{Co^{3+}}$.

(A24) $$\beta_{Co\;3\to 2}=\;\frac{1}{\delta -0.5}\;\cdot \;\frac{\left(1-2\delta \right)\cdot V_{Co^{3+}}+\left(2\delta -1\right)\cdot V_{Co^{2+}}}{2\cdot V_{Co^{3+}}}$$

(A25) $$\beta_{Co\;3\to 2}=\;\frac{1}{\delta -0.5}\;\cdot \;\frac{\left(0.5-\delta \right)\cdot V_{Co^{3+}}+\left(\delta -0.5\right)\cdot V_{Co^{2+}}}{V_{Co^{3+}}}$$

(A26) $$\beta_{Co\;3\to 2}=\;\frac{V_{Co^{2+}}-V_{Co^{3+}}\;}{V_{Co^{3+}}}$$

Equation (A24) is equivalent to Equation (A4) with *δ* = 1.5, and where all Co is in oxidation state 2+.

The volume of cobalt can be related to the cobalt ionic radius, which leads to Equation (A26).

(A27) $$\beta_{Co\;3\to 2}=\;\frac{r_{Co^{2+}}^{3}-r_{Co^{3+}}^{3}\;}{r_{Co^{3+}}^{3}}$$

### Appendix A.4. Chemical Expansion of Oxygen Vacancies Formation

The expression of the chemical expansion coefficient of oxygen vacancies is the same in both δ ranges and may be defined with the Equation (A28).

(A28) $$\beta_{v_{O}^{••}}=\;\frac{1}{\Delta \delta }\cdot \frac{\Delta V_{O}}{V_{O_{0}}}$$

As a *V_O_* is the average volume of oxygen site volume, which can also be calculated as weighted arithmetic means of oxygen ions and oxygen vacancies volume (Equation (A29)).

(A29) $$V_{O}=\;\frac{\left[O_{O}^{X}\right]\cdot V_{O_{O}^{X}}+\left[v_{O}^{••}\right]\cdot V_{V_{O}^{••}}}{6}$$

The molar concentration of oxygen vacancies is by definition equal to *δ*, thus the relation between the volume of oxygen site and *δ* can be written (Equation (A30)).

(A30) $$V_{O}=\;\frac{\left(6-\delta \right)\cdot V_{O_{O}^{X}}+\delta \cdot V_{V_{O}^{••}}}{6}$$

Substituting Equation (A27) to Equation (A25), the expression on the chemical expansion coefficient of oxygen vacancies is obtained (Equations (A31) and (A32)). The value of $\delta_{0}$ is now equivalent to *δ* at RT.

(A31) $$\beta_{v_{O}^{••}}=\;\frac{1}{\delta -\;\delta_{0}}\cdot \frac{\left(\delta_{0}-\;\delta \right)\cdot V_{O_{O}^{X}}\;+\left(\delta -\;\delta_{0}\right)\;\cdot V_{V_{O}^{••}}}{\left(6-\;\delta_{0}\right)\cdot V_{O_{O}^{X}}+\;\delta_{0}\cdot V_{V_{O}^{••}}}$$

(A32) $$\beta_{v_{O}^{••}}=\;\frac{\;V_{V_{O}^{••}\;}-\;V_{O_{O}^{X}}}{\left(6-\;\delta_{0}\right)\cdot V_{O_{O}^{X}}+\;\delta_{0}\cdot V_{V_{O}^{••}}}$$

Subtracting the calculated values of chemical expansion coefficient upon cobalt reduction from the total value, the chemical expansion coefficient of oxygen vacancies formation may be obtained; knowing the ionic radii of the oxygen ion, the size of oxygen vacancy can be determined.
