# Supplementary material for: High-Temperature Structural and Electrical Properties of BaLnCo2O6 Positrodes
Source: Materials (Basel). 2020 Sep 11;13(18):4044. doi: 10.3390/ma13184044 (PMC7558619; doi:10.3390/ma13184044)
Supplement: Supplementary file 1 [file materials-13-04044-s001.pdf]

Supplementary Information

High–Temperature Structural and Electrical Properties of Ba*Ln*Co_2O_6 Positrodes

Iga Szpunar [1,*], Ragnar Strandbakke [2,*], Magnus Helgerud Sørby [3], Sebastian Lech Wachowski [1], Maria Balaguer [4], Mateusz Tarach [4], José M. Serra [4], Agnieszka Witkowska [1], Ewa Dzik [1], Truls Norby [2], Maria Gazda [1] and Aleksandra Mielewczyk–Gryń [1,*]

[1] Nanotechnology Centre A, Faculty of Applied Physics and Mathematics and Advanced Materials Centre, Gdańsk University of Technology, ul. Narutowicza 11/12, 80−233 Gdańsk, Poland; sebastian.wachowski@pg.edu.pl (S.L.W.); agnieszka.witkowska@pg.edu.pl (A.W.); ewa.dzik@pg.edu.pl (E.D.); maria.gazda@pg.edu.pl (M.G.)

[2] Department of Chemistry, Centre for Materials Science and Nanotechnology, University of Oslo, FERMiO, Gaustadalléen 21, NO−0349 Oslo, Norway; truls.norby@kjemi.uio.no

[3] Department for Neutron Materials Characterization, Institute for Energy Technology, Instituttveien 18, 2007 Kjeller, Norway; magnus.sorby@ife.no

[4] Instituto de Tecnología Química, Universitat Politècnica de València, Consejo Superior de Investigaciones Científicas, Av. Naranjos s/n, E−46022 Valencia, Spain; mabara@upvnet.upv.es (M.B.); mata8@itq.upv.es (M.T.); jmserra@itq.upv.es (J.M.S.)

* Correspondence: iga.lewandowska@pg.edu.pl (I.S.); ragnar.strandbakke@kjemi.uio.no (R.S.); alegryn@pg.edu.pl (A.M.–G.)

Received: 7 July 2020; Accepted: 9 September 2020; Published: date

The oxygen vacancies concentration calculated from thermogravimetric oxidation studies was used for calculation the volumetric chemical expansion coefficient, however different heating rates were used in those two techniques. To eliminate the influence of this inconsistency, the additional oxidation studies were applied in the materials belonging to the investigated system, $BaGdCo_2O_{6-\delta}$. Figure S1. shows the oxygen stoichiometry as a function of temperature with material measured with different heating rate. There is no significant difference in temperature evolution of oxygen stoichiometry, thus, despite the experimental differences, these two techniques could have been combined.

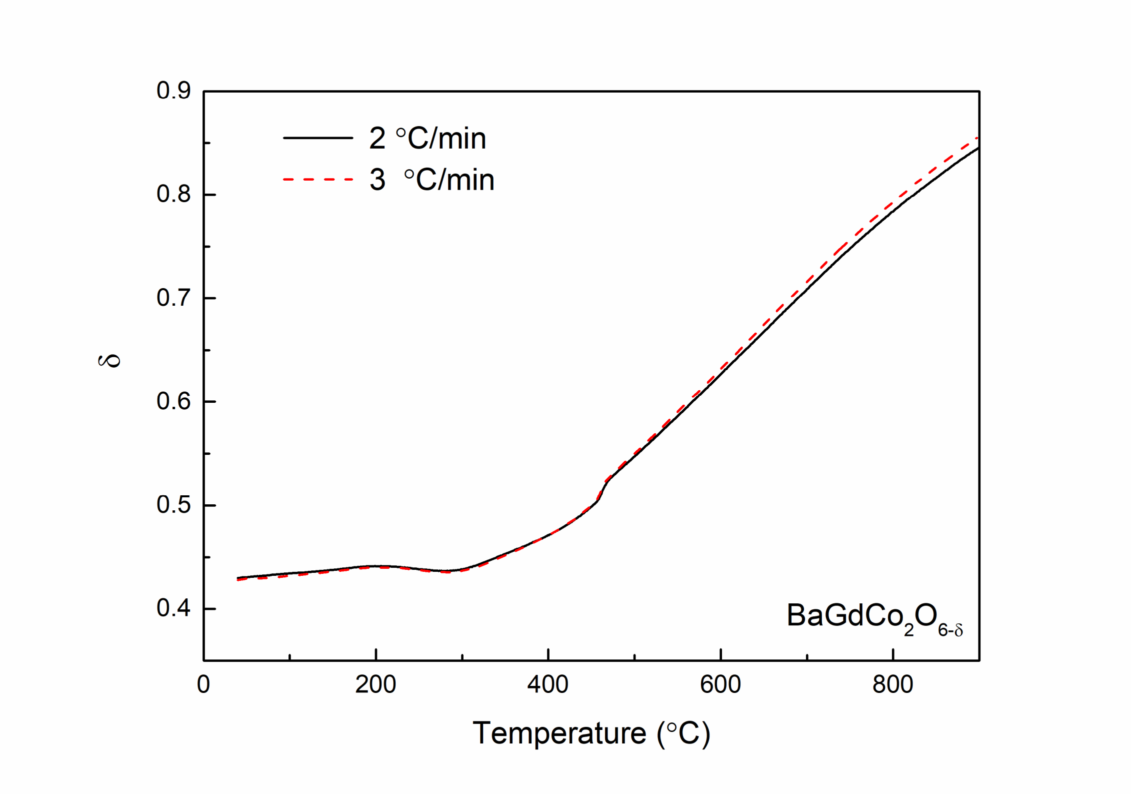

Figure S1. The temperature evolution of oxygen stoichiometry in $BaGdCo_2O_{6-\delta}$ with different heating rates.

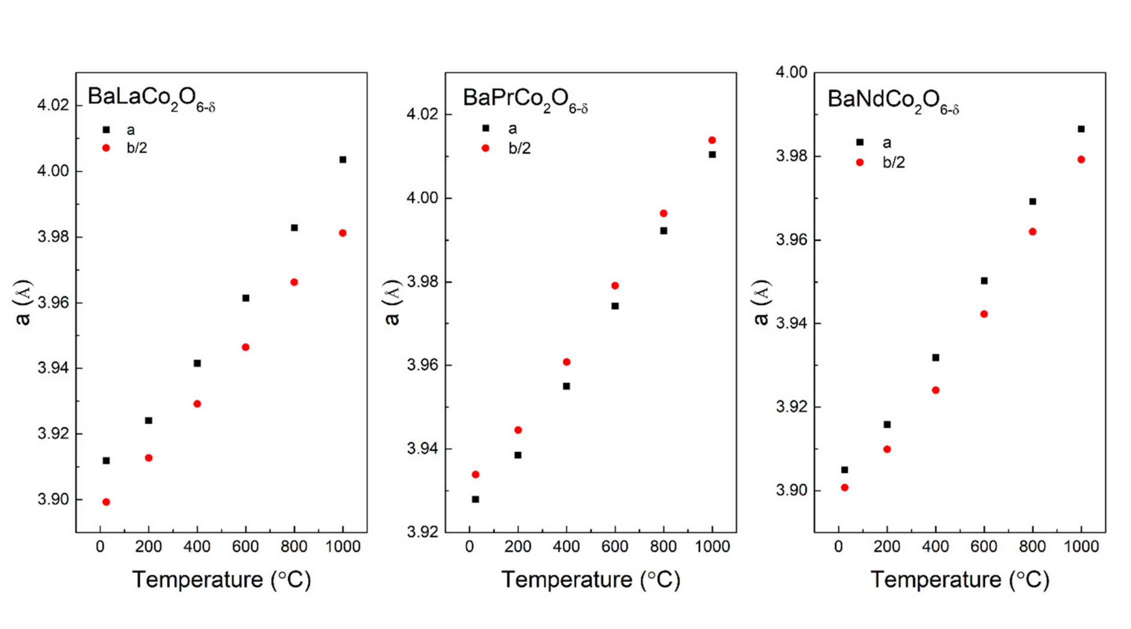

Figure S2. The temperature evolution of unit cell parameters a and b/2.

The supplementary information contains the detailed information on structure and the parameters describing the goodness of fit of the Rietveld Refinement.

Table S1. $BaLaCo_2O_{6-\delta}$ goodness of fit.

Temperature	Rwp	wR	R	R−bkg	wR−bkg	wRmin
25 °C	10.076 %	10.11 %	7.83 %	7.03 %	10.11 %	10.64 %
200 °C	12.579 %	10.71 %	8.28 %	7.51 %	10.71 %	12.91 %
400 °C	12.694 %	11.06 %	8.41 %	7.42 %	11.06 %	14.68 %
600 °C	11.884 %	10.69 %	8.23 %	7.42 %	10.69 %	14.60 %
800 °C	12.887 %	12.38 %	9.45 %	8.99 %	12.38 %	15.58%
1000 °C	26.515 %	32.24%	23.17%	32.17%	32.24%	17.70%

Table S2. Atomic coordinates and U_{iso} refined for orthorhombic phase of $BaLaCo_2O_{6-\delta}$. At room temperature.

**$BaLaCo_2O_{6-\delta}$**				
**25°C**				
**Pmmm**				
a (Å)	3.9119(2)			
b (Å)	7.7985(3)			
c (Å)	7.7116(3)			
	**x**	**y**	**z**	**U_{iso}**
La	$\frac{1}{2}$	0.247(2)	$\frac{1}{2}$	0.018(3)
Ba	$\frac{1}{2}$	0.248(2)	0	0.003(4)
Co1	0	$\frac{1}{2}$	0.258(3)	0.010(4)
Co2	0	0	0.245(3)	0.006(2)
O1	0	0	0	0.011(4)
O2	0	$\frac{1}{2}$	0	0.011(4)
O3	0	$\frac{1}{2}$	$\frac{1}{2}$	0.011(4)
O4	$\frac{1}{2}$	0	0.282(5)	0.011(4)
O5	$\frac{1}{2}$	$\frac{1}{2}$	0.345(5)	0.011(4)
O6	0	0.229(4)	0.254(5)	0.011(4)
O7	0	0	$\frac{1}{2}$	0.011(4)

Table S3. Atomic coordinates and U_{iso} refined for orthorhombic phase of $BaLaCo_2O_{6-\delta}$ at 200°C.

**$BaLaCo_2O_{6-\delta}$.**				
**200 °C**				
**Pmmm**				
a (Å)	3.9241(3)			
b (Å)	7.8255(5)			
c (Å)	7.7395(5)			
	**x**	**y**	**z**	**U_{iso}**
La	$\frac{1}{2}$	0.234(3)	$\frac{1}{2}$	0.038(2)
Ba	$\frac{1}{2}$	0.237(3)	0	0.028(2)
Co1	0	$\frac{1}{2}$	0.254(4)	0.042(4)

Co2	0	0	0.246(4)	0.017(4)
O1	0	0	0	0.011(4)
O2	0	$\frac{1}{2}$	0	0.011(4)
O3	0	$\frac{1}{2}$	$\frac{1}{2}$	0.011(4)
O4	$\frac{1}{2}$	0	0.282(5)	0.011(4)
O5	$\frac{1}{2}$	$\frac{1}{2}$	0.345(6)	0.011(4)
O6	0	0.229(5)	0.254(5)	0.011(4)
O7	0	0	$\frac{1}{2}$	0.011(4)

Table S4. Atomic coordinates and U_{iso} refined for orthorhombic phase of $BaLaCo_2O_{6-δ}$. At 400°C.

**$BaLaCo_2O_{6-δ}$**				
**400 °C**				
**Pmmm**				
a (Å)	3.941(2)			
b (Å)	7.858(1)			
c (Å)	7.769(1)			
	**x**	**Y**	**z**	**U_{iso}**
La	$\frac{1}{2}$	0.233(3)	$\frac{1}{2}$	0.0230
Ba	$\frac{1}{2}$	0.238(4)	0	0.0032
Co1	0	$\frac{1}{2}$	0.260(3)	0.0041
Co2	0	0	0.269(3)	0.0060
O1	0	0	0	0.011(4)
O2		$\frac{1}{2}$	0	0.011(4)
O3	0	$\frac{1}{2}$	$\frac{1}{2}$	0.011(4)
O4	$\frac{1}{2}$	0	0.282(6)	0.011(4)
O5	$\frac{1}{2}$	$\frac{1}{2}$	0.345(6)	0.011(4)
O6	0	0.229(5)	0.254(6)	0.011(4)
O7	0	0	$\frac{1}{2}$	0.011(4)

Table S5. Atomic coordinates and U_{iso} refined for orthorhombic phase of $BaLaCo_2O_{6-δ}$. At 600°C.

**$BaLaCo_2O_{6-δ}$**				
**600 °C**				
**Pmmm**				
a (Å)	3.9614(3)			
b (Å)	7.8927(3)			
c (Å)	7.7966(4)			
	**x**	**y**	**z**	**U_{iso}**
La	$\frac{1}{2}$	0.236(5)	$\frac{1}{2}$	0.032(7)
Ba	$\frac{1}{2}$	0.236(5)	0	0.017(7)

Co1	0	$\frac{1}{2}$	0.265(7)	0.020(9)
Co2	0	0	0.253(7)	0.012(9)
O1	0	0	0	0.011(4)
O2	0	$\frac{1}{2}$	0	0.011(4)
O3	0	$\frac{1}{2}$	$\frac{1}{2}$	0.011(4)
O4	$\frac{1}{2}$	0	0.282(6)	0.011(4)
O5	$\frac{1}{2}$	$\frac{1}{2}$	0.345(6)	0.011(4)
O6	0	0.229(5)	0.254(6)	0.011(4)
O7	0	0	$\frac{1}{2}$	0.011(4)

Table S6. Atomic coordinates and U_{iso} refined for orthorhombic phase of $BaLaCo_2O_{6-\delta}$. At 800°C.

**$BaLaCo_2O_{6-\delta}$**				
**800 °C**				
**Pmmm**				
a (Å)	3.982(1)			
b (Å)	7.932(2)			
c (Å)	7.830(2)			
	**x**	**y**	**z**	**U_{iso}**
La	$\frac{1}{2}$	0.237(5)	$\frac{1}{2}$	0.07(5)
Ba	$\frac{1}{2}$	0.242(4)	0	0.03(5)
Co1	0	$\frac{1}{2}$	0.237(9)	0.037(3)
Co2	0	0	0.242(9)	0.047(4)
O1	0	0	0	0.011(4)
O2	0	$\frac{1}{2}$	0	0.011(4)
O3	0	$\frac{1}{2}$	$\frac{1}{2}$	0.011(4)
O4	$\frac{1}{2}$	0	0.282(8)	0.011(4)
O5	$\frac{1}{2}$	$\frac{1}{2}$	0.345(7)	0.011(4)
O6	0	0.229(6)	0.254(8)	0.011(4)
O7	0	0	$\frac{1}{2}$	0.011(4)

Table S7. Atomic coordinates and U_{iso} refined for orthorhombic phase of $BaLaCo_2O_{6-\delta}$. At 1000°C.

**$BaLaCo_2O_{6-\delta}$**				
**1000 °C**				
**Pmmm**				
a (Å)	4.004(1)			
b (Å)	7.962(2)			
c (Å)	7.861(2)			
	**x**	**y**	**z**	**U_{iso}**
La	$\frac{1}{2}$	0.204(1)	$\frac{1}{2}$	0.06(1)

Ba	$\frac{1}{2}$	0.246(1)	0	0.06(1)
Co1	0	$\frac{1}{2}$	0.271(9)	0.05(3)
Co2	0	0	0.202(9)	0.06(3)
O1	0	0	0	0.011(4)
O2	0	$\frac{1}{2}$	0	0.011(4)
O3	0	$\frac{1}{2}$	$\frac{1}{2}$	0.011(4)
O4	$\frac{1}{2}$	0	0.282(8)	0.011(4)
O5	$\frac{1}{2}$	$\frac{1}{2}$	0.345(8)	0.011(4)
O6	0	0.229(6)	0.254(8)	0.011(4)
O7	0	0	$\frac{1}{2}$	0.011(4)

Table S8. Atomic coordinates and U_{iso} refined for tetragonal phase of $BaLaCo_2O_{6-\delta}$ at room temperature.

**$BaLaCo_2O_{6-\delta}$**				
**25°C**				
**P4/mmm**				
a (Å)	3.8734(1)			
c (Å)	7.7637(3)			
	**x**	**y**	**z**	**U_{iso}**
La	$\frac{1}{2}$	$\frac{1}{2}$	0	0.030(2)
Ba	$\frac{1}{2}$	$\frac{1}{2}$	$\frac{1}{2}$	0.035(3)
Co	0	0	0.250(2)	0.033(4)
O1	0	0	$\frac{1}{2}$	0.065(4)
O2	0	0	0	0.013(4)
O3	0	$\frac{1}{2}$	0.278(2)	0.016(8)

Table S9. Atomic coordinates and U_{iso} refined for tetragonal phase of $BaLaCo_2O_{6-\delta}$ at 200°C.

**$BaLaCo_2O_{6-\delta}$**				
**200°C**				
**P4/mmm**				
a (Å)	3.8864(2)			
c (Å)	7.7908(3)			
	**x**	**y**	**z**	**U_{iso}**
La	$\frac{1}{2}$	$\frac{1}{2}$	0	0.040(2)
Ba	$\frac{1}{2}$	$\frac{1}{2}$	$\frac{1}{2}$	0.044(2)
Co	0	0	0.251512	0.049(3)
O1	0	0	$\frac{1}{2}$	0.065(4)
O2	0	0	0	0.013(4)
O3	0	$\frac{1}{2}$	0.278099	0.016(8)

Table S10. Atomic coordinates and U_{iso} refined for tetragonal phase of $BaLaCo_2O_{6-\delta}$ at 400°C.

**$BaLaCo_2O_{6-\delta}$**				
**400°C**				
**P4/mmm**				
a (Å)	3.9010(3)			
c (Å)	7.8259(3)			
	**x**	**y**	**z**	**U_{iso}**
La	$\frac{1}{2}$	$\frac{1}{2}$	0	0.033(4)
Ba	$\frac{1}{2}$	$\frac{1}{2}$	$\frac{1}{2}$	0.040(5)
Co	0	0	0.252(4)	0.034(3)
O1	0	0	$\frac{1}{2}$	0.065(4)
O2	0	0	0	0.013(4)
O3	0	$\frac{1}{2}$	0.278(3)	0.016(8)

Table S11. Atomic coordinates and U_{iso} refined for tetragonal phase of $BaLaCo_2O_{6-\delta}$ at 600°C.

**$BaLaCo_2O_{6-\delta}$**				
**600°C**				
**P4/mmm**				
a (Å)	3.9165(3)			
c (Å)	7.8602(3)			
	**x**	**y**	**z**	**U_{iso}**
La	$\frac{1}{2}$	$\frac{1}{2}$	0	0.040(5)
Ba	$\frac{1}{2}$	$\frac{1}{2}$	$\frac{1}{2}$	0.038(5)
Co	0	0	0.246(3)	0.035(6)
O1	0	0	$\frac{1}{2}$	0.065(4)
O2	0	0	0	0.013(4)
O3	0	$\frac{1}{2}$	0.278(3)	0.016(8)

Table S12. Atomic coordinates and U_{iso} refined for tetragonal phase of $BaLaCo_2O_{6-\delta}$ at 800°C.

$BaLaCo_2O_{6-\delta}$				
800°C				
P4/mmm				
a (Å)	3.934(1)			
c (Å)	7.8973(3)			
	**x**	**y**	**z**	**U_{iso}**
La	$\frac{1}{2}$	$\frac{1}{2}$	0	0.043(5)
Ba	$\frac{1}{2}$	$\frac{1}{2}$	$\frac{1}{2}$	0.058(6)
Co	0	0	0.234(5)	0.0471
O1	0	0	$\frac{1}{2}$	0.065(4)
O2	0	0	0	0.013(4)

O3	0	$\frac{1}{2}$	0.278(3)	0.016(8)

Table S13. Atomic coordinates and U_{iso} refined for tetragonal phase of $BaLaCo_2O_{6-\delta}$ at 1000°C.

**$BaLaCo_2O_{6-\delta}$**				
**1000°C**				
**P4/mmm**				
a (Å)	3.951(1)			
c (Å)	7.9266(5)			
	**x**	**y**	**z**	**U_{iso}**
La	$\frac{1}{2}$	$\frac{1}{2}$	0	−0.036(6)
Ba	$\frac{1}{2}$	$\frac{1}{2}$	$\frac{1}{2}$	0.244(5)
Co	0	0	0.203(2)	0.032(5)
O1	0	0	$\frac{1}{2}$	0.065(4)
O2	0	0	0	0.013(4)
O3	0	$\frac{1}{2}$	0.278(3)	0.016(8)

Table S14. $BaPrCo_2O_{6-\delta}$ goodness of fit.

**Temperature**	**Rwp**	**wR**	**R**	**R−bkg**	**wR−bkg**	**wRmin**
25 °C	10.976 %	12.37%	9.11%	8.16%	12.37%	7.05%
200 °C	13.824 %	14.91%	10.63%	11.46%	14.91%	10.63%
400 °C	13.999 %	15.58%	11.46%	13.49%	15.58%	11.11%
600 °C	14.488 %	14.03%	11.09%	13.41%	14.03%	13.85%
800 °C	12.486 %	12.51%	9.42%	8.69%	12.51%	19.61%
1000 °C	23.313 %	23.45%	17.34%	22.15%	23.45%	21.74%

Table S15. Atomic coordinates and U_{iso} refined for orthorhombic phase of $BaPrCo_2O_{6-\delta}$ at room temperature.

**$BaPrCo_2O_{6-\delta}$**				
**25°C**				
**Pmmm**				
a (Å)	3.9271(5)			
b (Å)	7.864(1)			
c (Å)	7.6762(8)			
	**x**	**y**	**z**	**U_{iso}**
Pr	$\frac{1}{2}$	0.254(2)	$\frac{1}{2}$	0.012(1)
Ba	$\frac{1}{2}$	0.248(3)	0	−0.002(1)
Co1	0	$\frac{1}{2}$	0.245(3)	0.007(3)
Co2	0	0	0.250(1)	−0.002(3)
O1	0	0	0	0.040(7)
O2	0	$\frac{1}{2}$	0	−0.011(7)
O3	0	$\frac{1}{2}$	$\frac{1}{2}$	−0.014(7)

O4	$\frac{1}{2}$	0	0.275(4)	−0.028(7)
O5	$\frac{1}{2}$	$\frac{1}{2}$	0.249(5)	0.045(7)
O6	0	0.256(5)	0.287(4)	0.013(7)
O7	0	0	$\frac{1}{2}$	0.07(7)

Table S16. Atomic coordinates and U_{iso} refined for orthorhombic phase of $BaPrCo_2O_{6-\delta}$ at 200°C.

$BaPrCo_2O_{6-\delta}$				
**200°C**				
**Pmmm**				
a (Å)	3.9398(9)			
b (Å)	7.889(1)			
c (Å)	7.713(1)			
	**x**	**y**	**z**	U_{iso}
Pr	$\frac{1}{2}$	0.249(4)	$\frac{1}{2}$	0.009(2)
Ba	$\frac{1}{2}$	0.247(4)	0	−0.009(2)
Co1	0	$\frac{1}{2}$	0.245(1)	0.005(3)
Co2	0	0	0.252(1)	−0.010(3)
O1	0	0	0	0.09(1)
O2	0	$\frac{1}{2}$	0	−0.02(1)
O3	0	$\frac{1}{2}$	$\frac{1}{2}$	0.00(1)
O4	$\frac{1}{2}$	0	0.287(6)	0.08(2)
O5	$\frac{1}{2}$	$\frac{1}{2}$	0.267(5)	0.06(2)
O6	0	0.265(5)	0.287(4)	−0.05(2)
O7	0	0	$\frac{1}{2}$	−0.07(2)

Table S17. Atomic coordinates and U_{iso} refined for orthorhombic phase of $BaPrCo_2O_{6-\delta}$ at 400°C.

$BaPrCo_2O_{6-\delta}$				
**400°C**				
**Pmmm**				
a (Å)	3.956(1)			
b (Å)	7.921(1)			
c (Å)	7.744(1)			
	**x**	**y**	**z**	U_{iso}
Pr	$\frac{1}{2}$	0.250(7)	$\frac{1}{2}$	0.04(2)
Ba	$\frac{1}{2}$	0.248(7)	0	0.013(2)
Co1	0	$\frac{1}{2}$	0.25(1)	0.030(3)
Co2	0	0	0.25(1)	0.013(3)
O1	0	0	0	0.07(3)
O2	0	$\frac{1}{2}$	0	0.02(4)

O3	0	$\frac{1}{2}$	$\frac{1}{2}$	0.07(4)
O4	$\frac{1}{2}$	0	0.31(2)	0.02(4)
O5	$\frac{1}{2}$	$\frac{1}{2}$	0.29(2)	0.08(4)
O6	0	0.24(2)	0.27(2)	0.01(4)
O7	0	0	$\frac{1}{2}$	−0.05(4)

Table S18. Atomic coordinates and U_{iso} refined for orthorhombic phase of $BaPrCo_2O_{6-\delta}$ at 600°C.

**$BaPrCo_2O_{6-\delta}$**				
**600°C**				
**Pmmm**				
a (Å)	3.972(1)			
b (Å)	7.952(2)			
c (Å)	7.766(2)			
	**x**	**y**	**z**	**U_{iso}**
Pr	$\frac{1}{2}$	0.251(5)	$\frac{1}{2}$	0.048(3)
Ba	$\frac{1}{2}$	0.252(6)	0	0.029(3)
Co1	0	$\frac{1}{2}$	0.25(1)	0.031(5)
Co2	0	0	0.25(1)	0.037(5)
O1	0	0	0	−2.0(8)
O2	0	$\frac{1}{2}$	0	0.66(7)
O3	0	$\frac{1}{2}$	$\frac{1}{2}$	0.11(7)
O4	$\frac{1}{2}$	0	0.28(3)	0.09(7)
O5	$\frac{1}{2}$	$\frac{1}{2}$	0.28(3)	−0.01(7)
O6	0	0.26(2)	0.31(5)	0.03(7)
O7	0	0	$\frac{1}{2}$	−0.08(7)

Table S19. Atomic coordinates and U_{iso} refined for orthorhombic phase of $BaPrCo_2O_{6-\delta}$ at 800°C.

**$BaPrCo_2O_{6-\delta}$**				
**800°C**				
**Pmmm**				
a (Å)	3.990(1)			
b (Å)	7.988(1)			
c (Å)	7.795(1)			
	**x**	**y**	**z**	**U_{iso}**
Pr	$\frac{1}{2}$	0.251(1)	$\frac{1}{2}$	0.052(4)
Ba	$\frac{1}{2}$	0.248(1)	0	0.042(4)
Co1	0	$\frac{1}{2}$	0.25(2)	0.03(2)
Co2	0	0	0.25(2)	0.07(2)
O1	0	0	0	−0.02(4)

O2	0	$\frac{1}{2}$	0	−0.00(5)
O3	0	$\frac{1}{2}$	$\frac{1}{2}$	0.6(1)
O4	$\frac{1}{2}$	0	0.30(5)	−0.01(2)
O5	$\frac{1}{2}$	$\frac{1}{2}$	0.26(5)	0.13(2)
O6	0	0.23(4)	0.29(5)	0.07(2)
O7	0	0	$\frac{1}{2}$	0.4(1)

Table S20. Atomic coordinates and U_{iso} refined for orthorhombic phase of $BaPrCo_2O_{6-\delta}$ at 1000°C.

$BaPrCo_2O_{6-\delta}$				
**1000°C**				
**Pmmm**				
a (Å)	4.009(2)			
b (Å)	8.025(4)			
c (Å)	7.827(3)			
	**x**	**y**	**z**	U_{iso}
Pr	$\frac{1}{2}$	0.244(8)	$\frac{1}{2}$	0.003(2)
Ba	$\frac{1}{2}$	0.246(8)	0	0.011(2)
Co1	0	$\frac{1}{2}$	0.25(4)	0.04(4)
Co2	0	0	0.24(4)	−0.03(3)
O1	0	0	0	−0.07(5)
O2	0	$\frac{1}{2}$	0	−1.8(3)
O3	0	$\frac{1}{2}$	$\frac{1}{2}$	0.08(2)
O4	$\frac{1}{2}$	0	0.83(9)	−0.5(1)
O5	$\frac{1}{2}$	$\frac{1}{2}$	0.29(3)	0.5(1)
O6	0	0.22(5)	0.22(5)	−0.00(5)
O7	0	0	$\frac{1}{2}$	−0.42(7)

Table S21. $BaNdCo_2O_{6-\delta}$ goodness of fit.

Temperature	Rwp	wR	R	R−bkg	wR−bkg	wRmin
25 °C	16.52 %	16.52%	12.83%	14.34%	16.52%	12.41%
200 °C	15.953 %	15.95%	11.77%	11.77%	15.95%	12.16%
400 °C	16.761 %	16.76%	12.19%	14.04%	16.76%	13.45%
600 °C	16.625 %	16.62%	12.29%	14.00%	16.62%	14.36%
800 °C	15.106	15.11%	11.41%	13.15%	15.11%	15.76%
1000 °C	16.279 %	16.28%	12.26%	13.81%	16.28%	17.08%

Table S22. Atomic coordinates and U_{iso} refined for orthorhombic phase of $BaNdCo_2O_{6-δ}$ at room temperature.

**$BaNdCo_2O_{6-δ}$**				
**25°C**				
**Pmmm**				
a (Å)	3.905(1)			
b (Å)	7.801(3)			
c (Å)	7.656(2)			
	**x**	**y**	**z**	**U_{iso}**
Nd	$\frac{1}{2}$	0.253(3)	$\frac{1}{2}$	0.017(4)
Ba	$\frac{1}{2}$	0.251(3)	0	0.003(4)
Co1	0	$\frac{1}{2}$	0.246(2)	0.02(5)
Co2	0	0	0.253(5)	0.01(1)
O1	0	0	0	−0.02(7)
O2	0	$\frac{1}{2}$	0	−0.02(7)
O3	0	$\frac{1}{2}$	$\frac{1}{2}$	0.01(9)
O4	$\frac{1}{2}$	0	0.30(2)	−0.06(4)
O5	$\frac{1}{2}$	$\frac{1}{2}$	0.27(3)	−0.03(5)
O6	0	0.26(2)	0.28(2)	−0.01(3)
O7	0	0	$\frac{1}{2}$	−0.02(2)

Table S23. Atomic coordinates and U_{iso} refined for orthorhombic phase of $BaNdCo_2O_{6-δ}$ at 200°C.

**$BaNdCo_2O_{6-δ}$**				
**200°C**				
**Pmmm**				
a (Å)	3.916(1)			
b (Å)	7.819(2)			
c (Å)	7.682(1)			
	**x**	**y**	**z**	**U_{iso}**
Nd	$\frac{1}{2}$	0.251(2)	$\frac{1}{2}$	0.029(2)
Ba	$\frac{1}{2}$	0.248(2)	0	0.003(3)
Co1	0	$\frac{1}{2}$	0.224(2)	0.04(1)
Co2	0	0	0.246(3)	0.002(8)
O1	0	0	0	0.01(5)
O2	0	$\frac{1}{2}$	0	0.01(5)
O3	0	$\frac{1}{2}$	$\frac{1}{2}$	0.01(5)
O4	$\frac{1}{2}$	0	0.29(2)	0.01(5)
O5	$\frac{1}{2}$	$\frac{1}{2}$	0.27(2)	0.01(5)
O6	0	0.24(3)	0.28(2)	0.01(3)

O7	0	0	$\frac{1}{2}$	0.01(3)

Table S24. Atomic coordinates and U_{iso} refined for orthorhombic phase of $BaNdCo_2O_{6-δ}$ at 400°C.

**$BaNdCo_2O_{6-δ}$**				
**400°C**				
**Pmmm**				
a (Å)	3.932(1)			
b (Å)	7.848(2)			
c (Å)	7.711(2)			
	**x**	**y**	**z**	**U_{iso}**
Nd	$\frac{1}{2}$	0.255(1)	$\frac{1}{2}$	0.027(5)
Ba	$\frac{1}{2}$	0.250(2)	0	0.012(4)
Co1	0	$\frac{1}{2}$	0.238(4)	0.01(2)
Co2	0	0	0.268(4)	0.02(4)
O1	0	0	0	0.10(5)
O2	0	$\frac{1}{2}$	0	−0.05(6)
O3	0	$\frac{1}{2}$	$\frac{1}{2}$	0.09(6)
O4	$\frac{1}{2}$	0	0.30(5)	−0.08(6)
O5	$\frac{1}{2}$	$\frac{1}{2}$	0.28(5)	−0.03(6)
O6	0	0.26(6)	0.29(5)	0.07(6)
O7	0	0	$\frac{1}{2}$	−0.01(6)

Table S25. Atomic coordinates and U_{iso} refined for orthorhombic phase of $BaNdCo_2O_{6-δ}$ at 600°C.

**$BaNdCo_2O_{6-δ}$**				
**600°C**				
**Pmmm**				
a (Å)	3.950(2)			
b (Å)	7.885(2)			
c (Å)	7.739(3)			
	**x**	**y**	**z**	**U_{iso}**
Nd	$\frac{1}{2}$	0.253(4)	$\frac{1}{2}$	0.047(5)
Ba	$\frac{1}{2}$	0.249(3)	0	0.02(2)
Co1	0	$\frac{1}{2}$	0.233(3)	0.03(5)
Co2	0	0	0.258(3)	0.01(5)
O1	0	0	0	0.11(4)
O2	0	$\frac{1}{2}$	0	0.03(4)
O3	0	$\frac{1}{2}$	$\frac{1}{2}$	0.15(5)
O4	$\frac{1}{2}$	0	0.31(1)	−0.04(6)

O5	$\frac{1}{2}$	$\frac{1}{2}$	0.28(1)	−0.03(6)
O6	0	0.25(3)	0.30(1)	0.11(6)
O7	0	0	$\frac{1}{2}$	0.03(6)

Table S26. Atomic coordinates and U_{iso} refined for orthorhombic phase of $BaNdCo_2O_{6-\delta}$ at 800°C.

**$BaNdCo_2O_{6-\delta}$**				
**800°C**				
**Pmmm**				
a (Å)	3.970(2)			
b (Å)	7.924(3)			
c (Å)	7.7694(3)			
	**x**	**y**	**z**	**U_{iso}**
Nd	$\frac{1}{2}$	0.248(2)	$\frac{1}{2}$	0.091(6)
Ba	$\frac{1}{2}$	0.242(2)	0	0.054(5)
Co1	0	$\frac{1}{2}$	0.239(3)	0.069(9)
Co2	0	0	0.253(3)	0.067(9)
O1	0	0	0	0.09(6)
O2	0	$\frac{1}{2}$	0	0.01(4)
O3	0	$\frac{1}{2}$	$\frac{1}{2}$	0.10(4)
O4	$\frac{1}{2}$	0	0.325(7)	−0.07(5)
O5	$\frac{1}{2}$	$\frac{1}{2}$	0.250(8)	−0.07(5)
O6	0	0.26(1)	0.303(9)	0.12(4)
O7	0	0	$\frac{1}{2}$	0.02(4)

Table S27. Atomic coordinates and U_{iso} refined for orthorhombic phase of $BaNdCo_2O_{6-\delta}$ at 1000°C.

**$BaNdCo_2O_{6-\delta}$**				
**1000°C**				
**Pmmm**				
a (Å)	3.987(2)			
b (Å)	7.958(4)			
c (Å)	7.802(3)			
	**x**	**y**	**z**	**U_{iso}**
Nd	$\frac{1}{2}$	0.250(3)	$\frac{1}{2}$	0.085(9)
Ba	$\frac{1}{2}$	0.245(2)	0	0.047(8)
Co1	0	$\frac{1}{2}$	0.240(4)	0.07(1)
Co2	0	0	0.258(4)	0.03(1)
O1	0	0	0	0.3(1)
O2	0	$\frac{1}{2}$	0	−0.04(3)
O3	0	$\frac{1}{2}$	$\frac{1}{2}$	0.2(1)

O4	$\frac{1}{2}$	0	0.327(7)	−0.09(2)
O5	$\frac{1}{2}$	$\frac{1}{2}$	0.236(9)	−0.09(2)
O6	0	0.20(2)	0.30(1)	0.27(4)
O7	0	0	$\frac{1}{2}$	−0.19(7)

© 2020 by the authors. Submitted for possible open access publication under the terms and conditions of the Creative Commons Attribution (CC BY) license (http://creativecommons.org/licenses/by/4.0/).
